# Departures—Professional

**Published:** 1872-04

**Authors:** 


### EDITORIAL.

### Departures.—Professional.

Such is the appreciation of, and the demand for American dentists abroad, especially for those of ability and education, that quite a number of our younger members of the profession in this vicinity have gone and are going to foreign countries, to assume profitable and inviting positions, already prepared for them, and urging them to go and occupy.

Of those who have gone from this vicinity within a few years we may mention Dr. N. B. Slayton & Son, of Florence, Italy, whose efforts and labors during the entire time of their abode there (about ten years) have been crowned with very great success, as an evidence of which they have been compelled to call upon the profession here for aid.

Dr. H. H. Winn some six years ago went to Japan, and from there to Hong Kong, China, where he has been engaged in a very lucrative practice for over four years, and has established for himself a character and reputation of which any one might well be proud. He, too, calls upon the profession here for a co-laborer, which, from present indications, will ere long be sent to him.

Dr. J. G. Van Marter about five years ago went to Basle, Switzerland, and at once entered upon a lucrative practice. So great are professional demands upon him that he too has for sometime been loudly calling for help, which is now on the way to him in the person of Dr. C. M. Wright, late Professor of Mechanical Dentistry in the Ohio College of Dental Surgery. He takes with him his family, and expects to make Europe his home. So great and inviting are the inducements held out to him by Dr. Van Marter that he could not resist, though he had no special desire to leave his native country.

Dr. G. W. Field, who left this city about two years ago, has already made a good reputation and a very fine practice in Geneva, Switzerland.

Dr. Plotner, of Springfield, Ohio, has been in South America about three years, and has had success in practice far beyond his highest anticipations.

Dr. D. Phillips, of Springfield, Ohio, has gone to join Dr. Plotner

Such were the inducements held out to him as to cause him to leave a fine and remunerative practice in the city of his recent home.

Dr. N. W. Willlams, of Xenia, Ohio, within a few days leaves with his beautiful and accomplished bride for Florence, Italy, to join the Drs. Slayton, and will, without doubt, at once enter upon a most inviting field of professional labor.

In the departure of these gentlemen from our State the profession will lose in each of them an active, earnest worker. These men have all labored in various ways for the elevation of the profession, and their absence will be materially felt. But though they go from us, their influence may still be exercised even here for good. They can send us back their kind words of cheer, encouragement, and instruction, and this we trust they will not forget. Many others, whose names may be given hereafter, are making preparations to go within a few months to far distant countries, several of them being called by invitations quite as urgent as those to which reference has just been made.

These are all, with one exception, graduates of the Ohio Dental College.

Graduates of the other Dental Colleges of our country have gone and are still going to other countries to engage in the active duties of their profession. The present indications are that the Dental Colleges of the United States will, for the present at least, supply the world with efficient dentists. What an incentive to the colleges and what a stimulus to those who enter the profession.

